# Short Pulse Duration High-Power Laser Photocoagulation during Vitrectomy for Diabetic Retinopathy Reduces Postoperative Inflammation

**DOI:** 10.1371/journal.pone.0135126

**Published:** 2015-08-14

**Authors:** Masahiko Sugimoto, Atsushi Ichio, Mineo Kondo

**Affiliations:** Department of Ophthalmology, Mie University Graduate School of Medicine, Tsu, Japan; Bascom Palmer Eye Institute, University of Miami School of Medicine, UNITED STATES

## Abstract

The aim of this preliminary study was to determine the effectiveness of short pulse duration, high-power laser photocoagulation (PC) during vitrectomy for diabetic retinopathy (DR).The effects of short pulse duration PC with power of 340–360 mW and duration of 0.02 second were compared to conventional PC with power of 120–150 mW and duration of 0.2 second. The degree of inflammation was quantified by laser flare cell photometry before and at 1 day, 1 week, 4 week, and 12 weeks postoperatively. Twenty-two eyes of 22 consecutive patients were studied. Ten eyes were treated with short pulse duration PC and 12 eyes with conventional PC. The total energy was significantly lower in the short pulse duration PC group than in the conventional PC group (*P* = 0.007). The flare cell values were not significantly different between the two groups after 1 day, but at 1 week, the flare cell value was significantly lower in the short pulse duration PC group than in the conventional PC group (*P* = 0.04). This difference was also present at 4 and 12 weeks *(P<*0.05). The significant lower inflammation after short pulse duration PC than conventional PC indicates that the short pulse duration PC protocol should be considered to treat DR.

## Introduction

Panretinal photocoagulation (PRP) is a standard treatment for diabetic retinopathy (DR) since the two publications of the Diabetic Retinopathy Study and Early Treatment Diabetic Retinopathy Study established its effectiveness [1, 2]. The aim of PRP is to improve the ischemic status of the inner retina, and PRP has been demonstrated to have long-term efficacy. Vitrectomy is also a standard treatment for advanced DR, e.g., proliferative diabetic retinopathy (PDR), and it also leads to better visual outcomes [3, 4].

At present, diabetes mellitus (DM) is known to have an inflammatory component, and Williams and Nadler reported that immune-mediated inflammatory processes were involved in the pathophysiology of DR [5]. In addition, vascular leakage and non-perfused areas of the retina were found to be associated with retinal leukocyte stasis indicating that inflammation had developed in the retina [6].

Advancements in the vitrectomy technique for DR and PDR make it possible for good results, but there still remains severe complications related to inflammation. The inflammation begins immediately after the surgery which can then lead to many complications including the cell flare reflex, anterior hyaloidal fibrovascular proliferation, fibrovascular membrane formation, retinal detachment due to proliferative vitreous retinopathy, and rubeotic glaucoma [7]. Thus, it is important to reduce the degree of inflammation so that the number and severity of the complications after vitrectomy are ameliorated.

A disruption of the blood-ocular barrier develops under pathologic conditions which leads to leakage of serum proteins from the inflamed tissues. The degree of inflammation varies, and it is manifested by the presence of both cells and flare in the anterior chamber of the eye. It is important to quantify the amount of cells and flare accurately and objectively to be able to evaluate the degree of intraocular inflammation [8]. This can be done by a laser flare cell photometer which measures the degree of light scattering from a low-power He-Ne beam non-invasively [9]. The degree of inflammation has been determined in different ocular diseases and also before and after treatment [10–14]. Laser photocoagulation (PC) has been shown to be a successful way to treat moderate to advanced stages of DR. PC also affects the vitreous levels of cytokines in eyes with PDR [15] and aqueous cell flare [16].

A new laser PC techniques, called pattern scan laser PC, was recently developed by Topcon Medical Laser Systems (Santa Clara, CA, USA). Pattern scan laser can deliver multiple laser burns in the form of a pattern array with a single depression of a foot pedal. This technique also delivers less thermal energy with the use of short pulse duration high-power laser PC, and the degree of tissue damage is less than that following conventional PC. This system is currently used widely for various retinal vascular diseases [17–20], and its safety and efficacy have been reported [21]. There is a possibility that the reduction in inflammation resulted in fewer complications after PRP [22].

Although the potential advantages of short pulse duration laser PC are well known, it has not been determined whether this technique can be applied for PC during vitrectomy. We hypothesized that short pulse duration laser PC during vitrectomy will reduce the degree of inflammation and complications. To test this hypothesis, we compared the degree of inflammation before and after short pulse duration laser PC to those after conventional laser PC in patients with DR.

## Patients and Methods

### Patients

This was a prospective, non-randomized study, and the procedures were approved by the Institutional Ethics Review Board of the Mie University Hospital (#2678), and all patients provided an oral and signed informed consent. The procedures used adhered to the tenets of the Declaration of Helsinki.

Twenty-two eyes from 22 consecutive DR patients who required vitrectomy served as subjects. The patients were examined between from April to September of 2013. The estimated glomerular filtration rate (eGFR; 60–120 ml/min/m^2^) represents the renal function and the HbA1c (4.6–6.2%) reflects the control of diabetes. Patients with glaucoma, uveitis, exfoliation syndrome, previous ocular disease, and those receiving topical or systemic anti-inflammatory therapies were excluded. The 22 eyes included 9 with vitreous hemorrhage (VH), 7 with diabetic macular edema (DME), and 6 with PDR requiring vitrectomy. Based on the clinical DR disease severity scales, 15 eyes were classified as PDR (9 of the VH and 6 of the PDR eyes), while 7 eyes were classified as moderate nonproliferative DR (7 of the DME eyes). Twenty of 22 eyes had already had panretinal photocoagulation before the vitrectomy. There were five eyes with a retinal detachment before the surgery. Each eye was placed into the short pulse duration PC group or the conventional PC group in a nonrandomized manner determined by one physician (MS). The demographic data of the two groups are shown in Table 1.

**Table 1:** Demographic data of short pulse duration PC group and conventional PC group.

	Short pulse duration PC group	Conventional PC group	P value
No. of eyes	10	12	
Age (yrs)	67.7 ± 4.4	64.8 ± 4.6	0.67
Duration of DM (yrs)	18.3 ± 13.3	12.3±10.4	0.25
eGFR	49.7 ± 29.4	47.3 ± 22.0	0.83
HbA1c (%)	7.86 ± 1.10	7.42 ± 1.30	0.40
Previous PRP (+/-)	8/2	12/0	0.10
Reason for surgery			
VH	5	4	
DME	3	4	
PDR	2	4	0.40
Presence of RD (+/-)	2/8	3/9	0.78
Duration of surgery (min.)	105.4 ± 40.3	128.4 ± 61.6	0.03*
23 gauge/25 gauge	0/10	1/11	0.35
Combined PEA+IOL (+/-)	5/5	9/3	0.22
Tamponade (+/-)	1/9	5/7	0.10

Data are shown as the mean ± standard deviation. Unpaired t-test or Chi-squared test was used to compare between the groups. eGFR, estimated glomerular filtration rate; PRP, pan-retinal photocoagulation; VH, vitreous hemorrhage; DME, diabetic macular edema; PDR, proliferative diabetic retinopathy; RD, retinal detachment; PEA + IOL, phacoemulsification and IOL implantation.

*P < 0.05.

One surgeon (MS) performed all surgeries. After local anesthesia by a sub-Tenon capsule injection of idocaine hydrochloride, a standard 4-port pars plana vitrectomy was performed using a 23-gauge or 25-gauge microincision procedures with chandelier illumination by a single vitrectomy system (Constellation Vision System, Alcon, Irvine, CA, USA). All the patients underwent basically the same procedure except for the treatment of laser photocoagulation. After the core vitrectomy, the posterior hyaloid membrane was completely removed which released all retinal traction. Proliferative membranes were removed if present.

All patients received PC during the vitrectomy with 10 eyes receiving short pulse duration laser PC and 12 eyes receiving conventional laser PC. Eyes were injected with gaseous or silicon oil tamponade if a retinal detachment was present.

Phacoemulsification and IOL implantation were performed prior to the pars plana vitrectomy but only on those patients >50-years-of-age and those with a cataract that prevented a clear view of the retina during surgery. After the surgery, all patients received prophylactic topical antibiotics, steroidal and non-steroidal anti-inflammatory drugs (NSAIDs) for 1 month.

### Photocoagulation (PC) parameters

**Fig 1:**
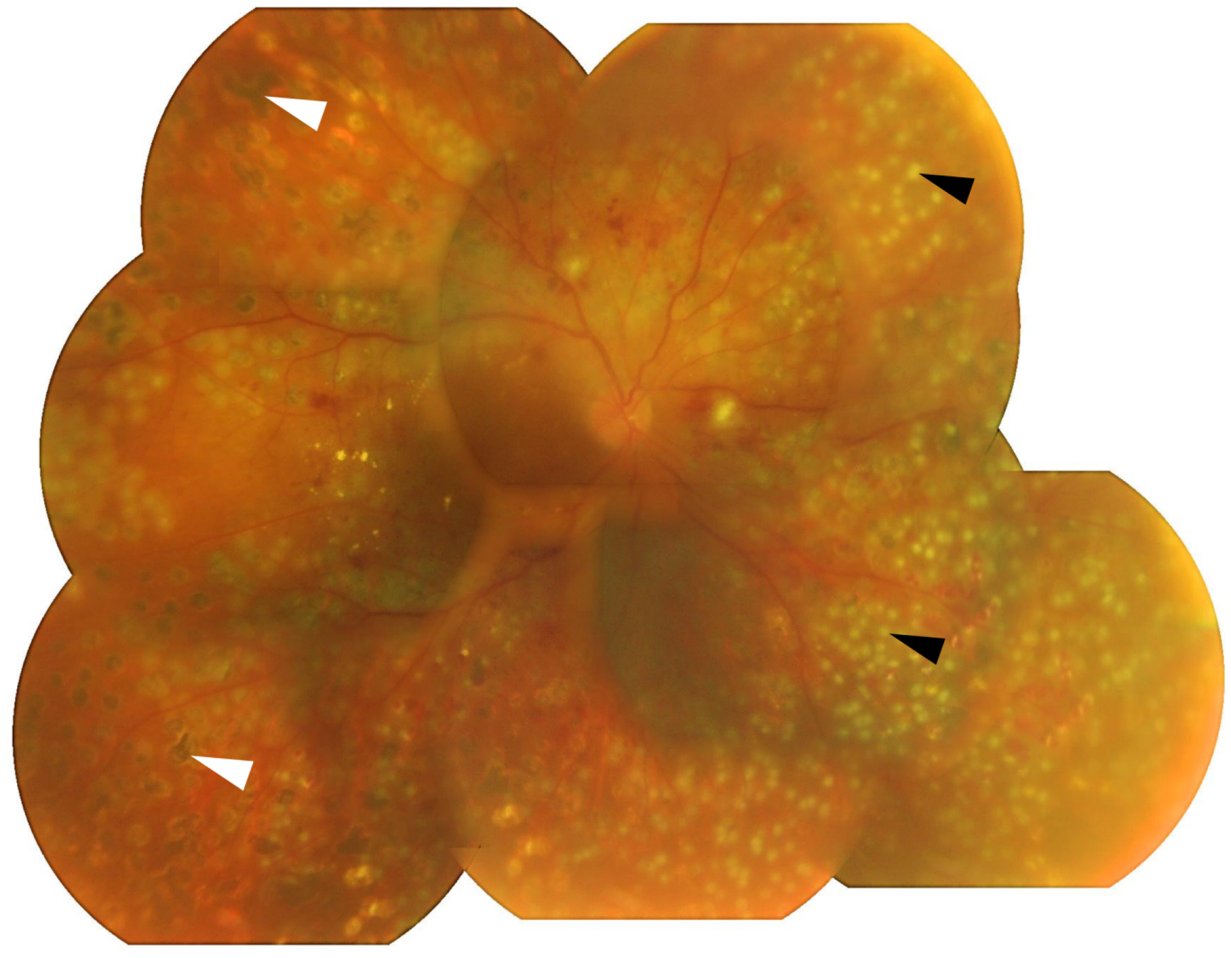
Fundus photograph of eye after short pulse duration high-power laser PC on postoperative day 1. Small PC spots of short pulse duration high power laser PC are seen (black arrow heads). Spot size during surgery was determined to be strong enough to cause a white spot. The degenerated spots are due to photocoagulation created at an outpatient clinic before being examined in our clinic (white arrow heads).

Laser PC during vitrectomy surgery was performed with a laser system (Purepoint, 532 nm) which is equipped with a standard Constellation Vision System (Alcon, Irvine, CA, USA). For short pulse duration PC, the laser power setting was 340–360 mW, duration was 0.02 seconds, and the interval was 0.2 seconds. For conventional PC, the laser power setting was 120–150 mW, the duration was 0.2 seconds, and the interval was 0.2 seconds. Because different PC instruments were used in the outpatient clinic, it is difficult to determine the exact size of the PC created during vitrectomy. However in all cases, the laser power was set strong enough to cause a white spot to appear on the retina. The appearance of the short pulse laser spots after surgery are shown in Fig 1. These two laser during surgery were also shown in S1 Movie. The total PC energy was automatically calculated by the embedded software.

### Laser flare cell photometry

A laser flare cell photometer (FM-600, Kowa, Nagoya, Japan) was used to quantify the degree of aqueous flare and cells according to the manufacturer’s protocol. Laser flare cell measurements were performed preoperatively and at 1 day, 1 week, 4 weeks, and 12 weeks postoperatively. The average of 5 measurements at each visit was defined as the cell flare count.

### Statistical analyses

Data are expressed as the means ± standard deviations. Chi-squared tests were used to determine the significance of differences between the two groups. Unpaired *t* tests were used to determine the significance of differences between the short pulse duration PC group and the conventional PC group. All statistical tests were 2-sided with *P* values <0.05 accepted to be significant.

## Results

At the baseline, there were no significant differences in the age, duration of the DM, the presence of previous panretinal photocoagulation, reason for surgery, presence of retinal detachment between the two groups. *(P* >0.05, Table 1). Although it has been previously reported that severe renal dysfunction or poor control of DM is related to the onset of post-operative inflammation or complications [23], we found no significant difference between our two groups. There was also no significant difference in the number of 23-gauge and 25-gauge vitrectomy surgeries and in the number of combined phacoemulsification and IOL implantation. The rate of tamponade use was also not significantly different. However, the duration of surgery was significantly shorter in the short pulse duration PC group (105.4 ± 40.3 min.) than in the conventional PC group (128.4 ± 61.6 min., *P* = 0.03, Table 1).

The mean number of laser PC shots in the short pulse duration PC group (2830 ± 635) was significantly higher than in the conventional PC group (1951 ± 813, *P* = 0.03, Table 2). On the other hand, the total PC energy during the surgery in the short pulse duration PC group (25.4 ± 20.8 mJ) was significantly lower than in the conventional PC group (54.7 ± 26.7 mJ, *P* = 0.007).

**Table 2:** Data of laser photocoagulation (PC) in short pulse duration PC group and conventional PC group during surgery.

	Short pulse duration PC group	Conventional PC group	*p* value
No. of PC shots	2830 ± 635	1951 ± 813	0.03*
Total energy (mJ)	25.4 ± 20.8	54.7 ± 26.7	0.007*

Data are expressed as the means ± standard deviations. Unpaired t-tests were used to determine the significance of differences between two groups.

*P < 0.05.

**Fig 2:**
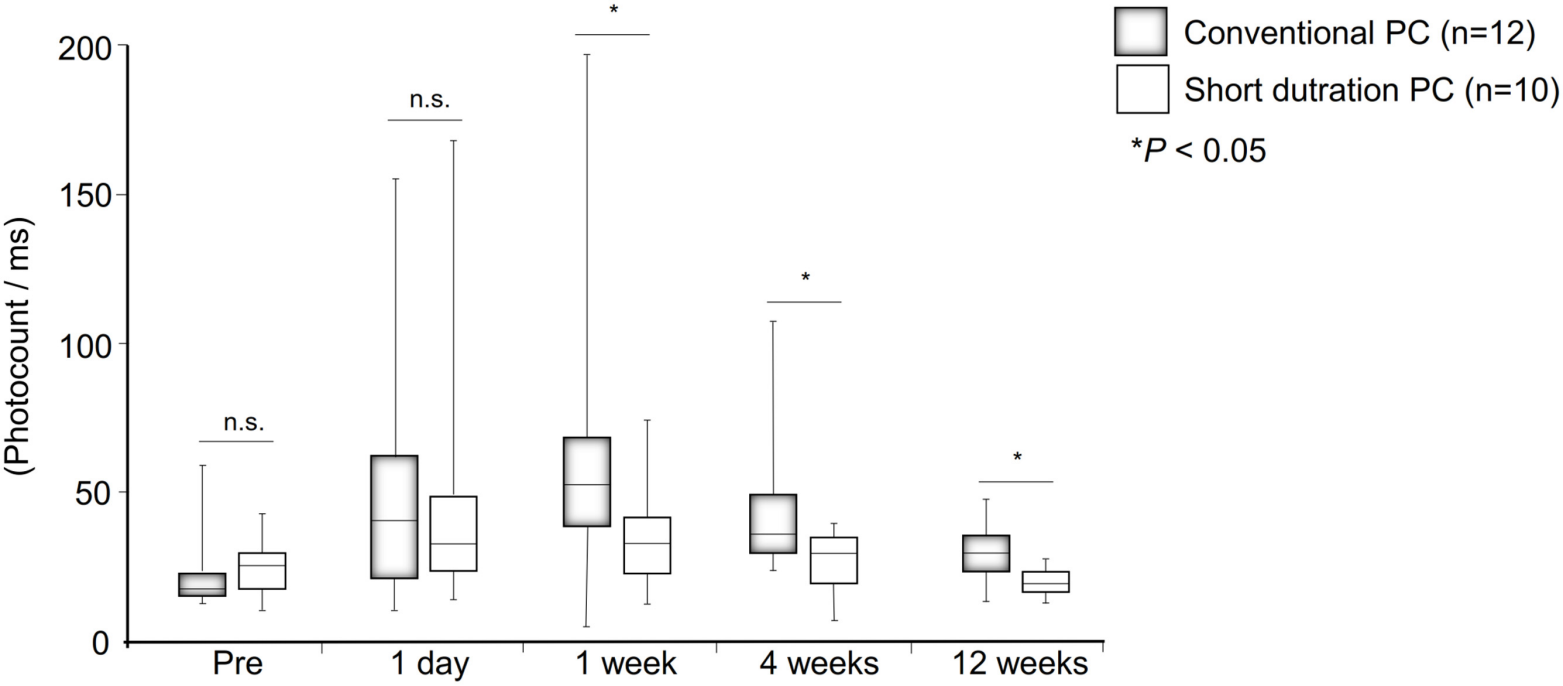
Laser flare cell values before, and at 1 day, 1 week, 4 week, and 12 weeks postoperatively for the short pulse duration PC group and the conventional PC group. The laser flare cell values were significantly lower in the short pulse duration PC group than in the conventional PC group at 1 week, 4 weeks and 12 weeks *(P <*0.05). Data are presented as Box–whisker plot and analyzed with Unpaired *t* tests.

No significant differences in the flare cell values were observed preoperatively in the two groups *(P* = 0.83, Fig 2). One day after surgery, the flare cell values were increased in both groups, but the difference was not significant *(P* = 0.89). At 1 week, the flare cell values were significantly lower in the short pulse duration PC group (*P* = 0.04), and this difference remained at 4 weeks *(P* = 0.04) and 12 weeks *(P* = 0.03).

None of the eyes in either group had any signs of iris or retinal neovascularization during the follow-up of 12 weeks after the surgery. A recurrence and absorbance of the VH was seen in 1 eye in the short duration PC group, but the VH resolved after a second surgery at 4 months after the primary surgery. No obvious signs of the existence of neovascularization were observed during the second surgery.

## Discussion

**T**he main purpose of PC in eyes with DR is to reduce the ischemic status of the inner retina by destroying the retinal pigment epithelium. This results in a reduction of VEGF secretion and improvement of the inadequate oxygen supply. However, excessive PC during the vitrectomy often causes postoperative inflammation and complications such as macular edema and posterior or anterior synechiae [24]. Thus, it is essential to minimize the postoperative inflammation associated with intraoperative PC during the vitrectomy. Chemical inflammatory mediators associated with vitrectomy can be reduced by oral or topical medications such as corticosteroids or NSAIDs, but it is still difficult to completely prevent the inflammation. It is likely that the decrease in the number of PC shots during the surgery can reduce inflammation, however there still remains the risk of the progression of the neovascularization such as rubeotic glaucoma.

It was recently reported that short pulse duration high-power PC used in the pattern scan laser PC system can reduce such inflammation and lead to effective treatment [25 26]. This technique delivers less thermal energy compared to conventional PC, and results in less inflammation and damage to both the outer retina and retinal pigment epithelium. Thus, the complications should be reduced after short pulse duration high-power pattern scan laser PC [22]. However as best we know, no study has been published comparing the post-PC inflammation between conventional PC and short pulse duration high-power PC for clinical use. Our results showed that the degree of inflammation was lower after short pulse duration than after conventional PC for clinical use.

Because the currently available pattern scan PC devices were not made for intraoperative PC, we used the laser system which is part of the standard vitrectomy system. We found that even though the number of total PC was significantly higher in the short pulse duration PC group, the total PC energy during surgery was significantly lower in the short duration PC group than that of the conventional PC group (Table 2). In addition, the flare cell values were significantly lower in the short pulse duration PC group than in the conventional PC group at 1, 4, and 12 weeks (Fig 2). Many studies have reported the development of postoperative inflammation for various diseases, and they reported that the peak flare value occurred on day 1 [10–14]. We found the same with the conventional PC group. In addition, there was no significant difference between flare value on day 1 and 1 week with conventional PC (*P* >0.05), and there is no report on the time course of flare cell changes after vitrectomy for DR. So there is a possibility that DR eyes may have a longer period of inflammation compared to other diseases.

Because six of 22 patients had gas or silicon oil tamponade, we also compared the flare cell values only in patients whose vitrectomy was performed without tamponade. We found that the flare cell values still tended to be lower in the short pulse duration PC group at all-time points, but the values were significantly lower only at 12 weeks (*P* <0.05, data not shown). These results suggest that there is a possibility that the short pulse duration PC protocol may be one of the options to reduce the inflammation caused by intraoperative PC.

Although our study showed the usefulness of short pulse duration PC during the vitrectomy, some problems remain. One of our cases that had been treated with short pulse duration PC had a recurrence of a VH. The recurrence developed after the absorbance of the VH after the initial PC, and the patient had to undergo a second surgery 4 months later. We noted during the second surgery that the PC scars in this eye were weak and could be hardly detected during the second operation. Therefore, we applied additional PC using the conventional PC technique during the second surgery. Similar problems have been reported in eyes treated with the commercial PASCAL system, and the authors concluded that the pattern scan laser was not satisfactory to control the ischemic changes [27].

Our study has some of major limitations. First, our study was not randomized for subject assignment and the number of eyes was small. There were no significant differences in the demographics of the two groups, and PDR cases were included in both groups. So there may be a possibility that the severity of PDR was related with their differences with the operation duration. In addition, flare cell values or aqueous protein concentrations are also increased in eyes with advanced stages of DR [28, 29]. To resolve these problem, we have to select patients much strictly. To avoid stress during surgery, i.e., proliferative membrane manipulations, except influence of PC, participants must be limited to DME or simple VH. This was because we have performed this trial as a preliminary study to evaluate the usefulness and safety of short pulse duration PC on the postoperative inflammation. However, given the results, we believe that a larger, prospective study is warranted to further investigate this issue.

In conclusion, we studied the effects of short pulse duration high-power PC on the aqueous flare values after vitrectomy for DR. The inflammation was significantly lower than that following conventional PC. Our preliminary study shows that short pulse duration PC may be a technique that allows for less inflammation during vitrectomy for DR using available instruments and will reduce complications.

## Supporting Information

S1 Movie Conventional and short pulse duration PC during surgery. (MP4)
